# Supplementary material for: An autoimmune pleiotropic SNP modulates IRF5 alternative promoter usage through ZBTB3-mediated chromatin looping
Source: Nat Commun. 2023 Mar 3;14:1208. doi: 10.1038/s41467-023-36897-z (PMC9984425; doi:10.1038/s41467-023-36897-z)
Supplement: Supplementary file 1 — Supplementary Information [file 41467_2023_36897_MOESM1_ESM.pdf]

Supplementary Information

An autoimmune pleiotropic SNP modulates *IRF5* alternative promoter usage through ZBTB3-mediated chromatin looping

This file includes:
Supplementary Figures S1 to S11

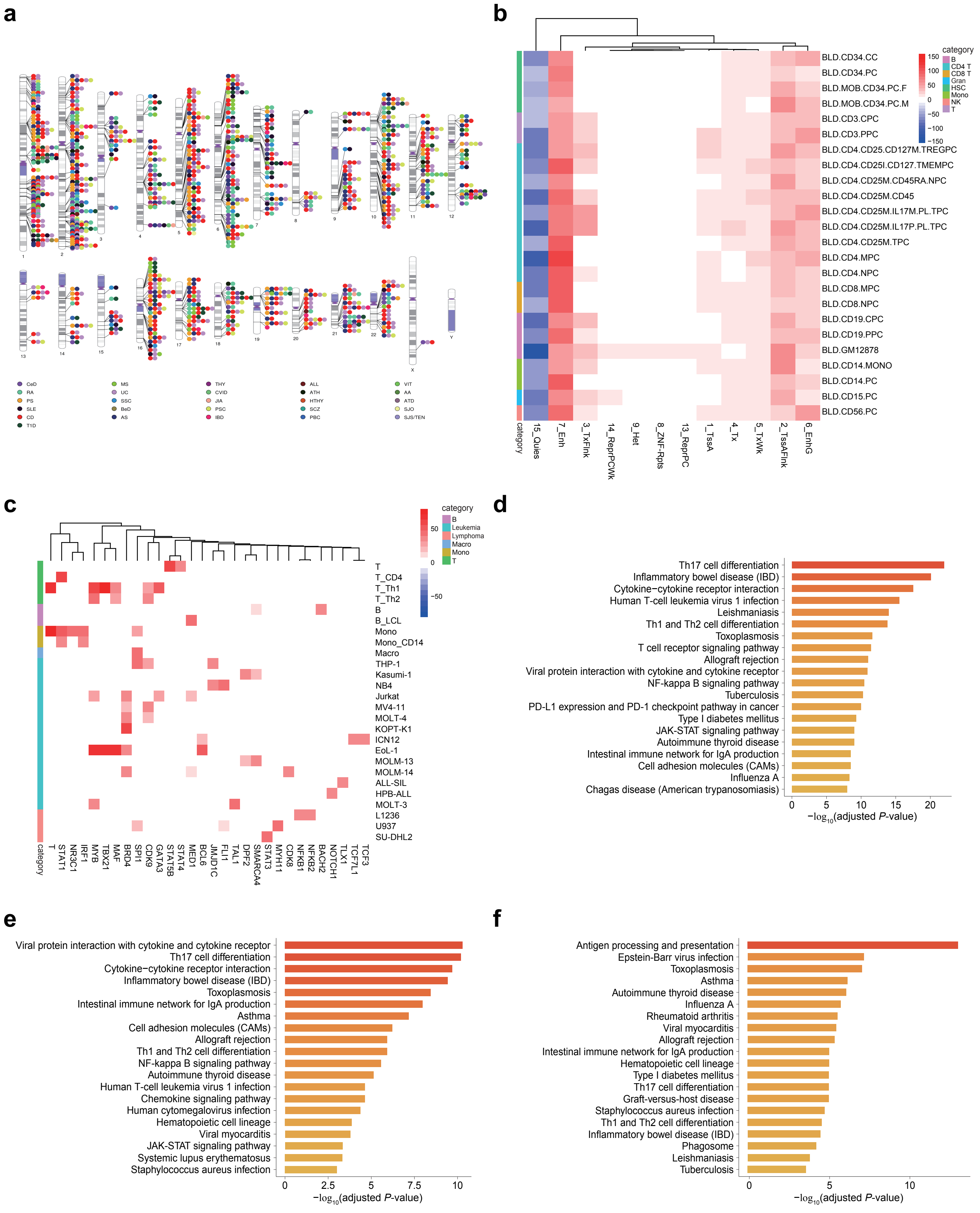
a
CeD
RA
PS
SLE
CD
T1D
MS
UC
SSC
BeD
AS
THY
CVID
JIA
PSC
IBD
ALL
ATH
HTHY
SCZ
PBC
VIT
AA
ATD
SJO
SJS/TEN
b
category
B
CD4 T
CD8 T
Gran
HSC
Mono
NK
T
150
100
50
0
−50
−100
−150
BLD.CD34.CC
BLD.CD34.PC
BLD.MOB.CD34.PC.F
BLD.MOB.CD34.PC.M
BLD.CD3.CPC
BLD.CD3.PPC
BLD.CD4.CD25.CD127M.TREGPC
BLD.CD4.CD25I.CD127.TMEMPC
BLD.CD4.CD25M.CD45RA.NPC
BLD.CD4.CD25M.CD45
BLD.CD4.CD25M.IL17M.PL.TPC
BLD.CD4.CD25M.IL17P.PL.TPC
BLD.CD4.CD25M.TPC
BLD.CD4.MPC
BLD.CD4.NPC
BLD.CD8.MPC
BLD.CD8.NPC
BLD.CD19.CPC
BLD.CD19.PPC
BLD.GM12878
BLD.CD14.MONO
BLD.CD14.PC
BLD.CD15.PC
BLD.CD56.PC
category
15_Quies
7_Enh
3_TxFlnk
14_ReprPCWk
9_Het
8_ZNF-Rpts
13_ReprPC
1_TssA
4_Tx
5_TxWk
2_TssAFlnk
6_EnhG
c
category
B
Leukemia
Lymphoma
Macro
Mono
T
50
0
−50
T
T_CD4
T_Th1
T_Th2
B
B_LCL
Mono
Mono_CD14
Macro
THP-1
Kasumi-1
NB4
Jurkat
MV4-11
MOLT-4
KOPT-K1
ICN12
EoL-1
MOLM-13
MOLM-14
ALL-SIL
HPB-ALL
MOLT-3
L1236
U937
SU-DHL2
category
T
STAT1
NR3C1
IRF1
MYB
TBX21
MAF
BRD4
SPI1
CDK9
GATA3
STAT5B
STAT4
MED1
BCL6
JMJD1C
FLI1
TAL1
DPF2
SMARCA4
STAT3
MYH11
CDK8
NFKB1
NFKB2
BACH2
NOTCH1
TLX1
TCF7L1
TCF3
d
Th17 cell differentiation
Inflammatory bowel disease (IBD)
Cytokine−cytokine receptor interaction
Human T-cell leukemia virus 1 infection
Leishmaniasis
Th1 and Th2 cell differentiation
Toxoplasmosis
T cell receptor signaling pathway
Allograft rejection
Viral protein interaction with cytokine and cytokine receptor
NF-kappa B signaling pathway
Tuberculosis
PD-L1 expression and PD-1 checkpoint pathway in cancer
Type I diabetes mellitus
JAK-STAT signaling pathway
Autoimmune thyroid disease
Intestinal immune network for IgA production
Cell adhesion molecules (CAMs)
Influenza A
Chagas disease (American trypanosomiasis)
0
5
10
15
20
−log10(adjusted P-value)
e
Viral protein interaction with cytokine and cytokine receptor
Th17 cell differentiation
Cytokine−cytokine receptor interaction
Inflammatory bowel disease (IBD)
Toxoplasmosis
Intestinal immune network for IgA production
Asthma
Cell adhesion molecules (CAMs)
Allograft rejection
Th1 and Th2 cell differentiation
NF-kappa B signaling pathway
Autoimmune thyroid disease
Human T-cell leukemia virus 1 infection
Chemokine signaling pathway
Human cytomegalovirus infection
Hematopoietic cell lineage
Viral myocarditis
JAK-STAT signaling pathway
Systemic lupus erythematosus
Staphylococcus aureus infection
0
2.5
5
7.5
10
−log10(adjusted P-value)
f
Antigen processing and presentation
Epstein-Barr virus infection
Toxoplasmosis
Asthma
Autoimmune thyroid disease
Influenza A
Rheumatoid arthritis
Viral myocarditis
Allograft rejection
Intestinal immune network for IgA production
Hematopoietic cell lineage
Type I diabetes mellitus
Th17 cell differentiation
Graft-versus-host disease
Staphylococcus aureus infection
Th1 and Th2 cell differentiation
Inflammatory bowel disease (IBD)
Phagosome
Leishmaniasis
Tuberculosis
0
5
10
−log10(adjusted P-value)

Supplementary Fig. 1 | Functional annotation and target gene analysis of autoimmune disease-associated pleiotropic variants. a Genome-Wide Association Studies (GWAS) diagram shows the curated potential pleiotropic or shared genetic loci associated with different autoimmune diseases. Numbers and letters mark chromosome order numbers. Abbreviations for autoimmune diseases: celiac disease (CeD), rheumatoid arthritis (RA), psoriasis (PS), systemic lupus erythematosus (SLE), Crohn's disease (CD), type 1 diabetes (T1D), multiple sclerosis (MS), ulcerative colitis (UC), systemic sclerosis (SSC), Behcet's disease (BeD), ankylosing spondylitis (AS), thyroiditis (THY), common variable immunodeficiency (CVID), juvenile idiopathic arthritis (JIA), primary sclerosing cholangitis (PSC), inflammatory bowel disease (IBD), any allergies (ALL), asthma (ATH), hypothyroidism (HTHY), schizophrenia (SCZ), primary biliary cirrhosis (PBC), vitiligo (VIT), alopecia areata (AA), autoimmune thyroid disease (ATD), Sjogren's syndrome (SJO), and Stevens-Johnson syndrome/toxic epidermal necrolysis (SJS/TEN). **b**, **c** Enrichment analysis of the pleiotropic variants in certain chromatin states (**b**) or transcription factor (TF) binding (**c**) for specific blood cell types. MONO: monocytes. **d**–**f** Kyoto Encyclopedia of Genes and Genomes (KEGG) pathway enrichment analysis for the DEPICT-identified target genes affected by the pleiotropic variants (**d**), Expression Quantitative Trait Locus (eQTL) Regulatory Trait Concordance (RTC) genes associated with the pleiotropic variants (**e**), and Promoter Capture Hi-C (PCHi-C) genes linked to the pleiotropic variants (**f**), and over-representation test is used to calculate adjusted *P*-value.

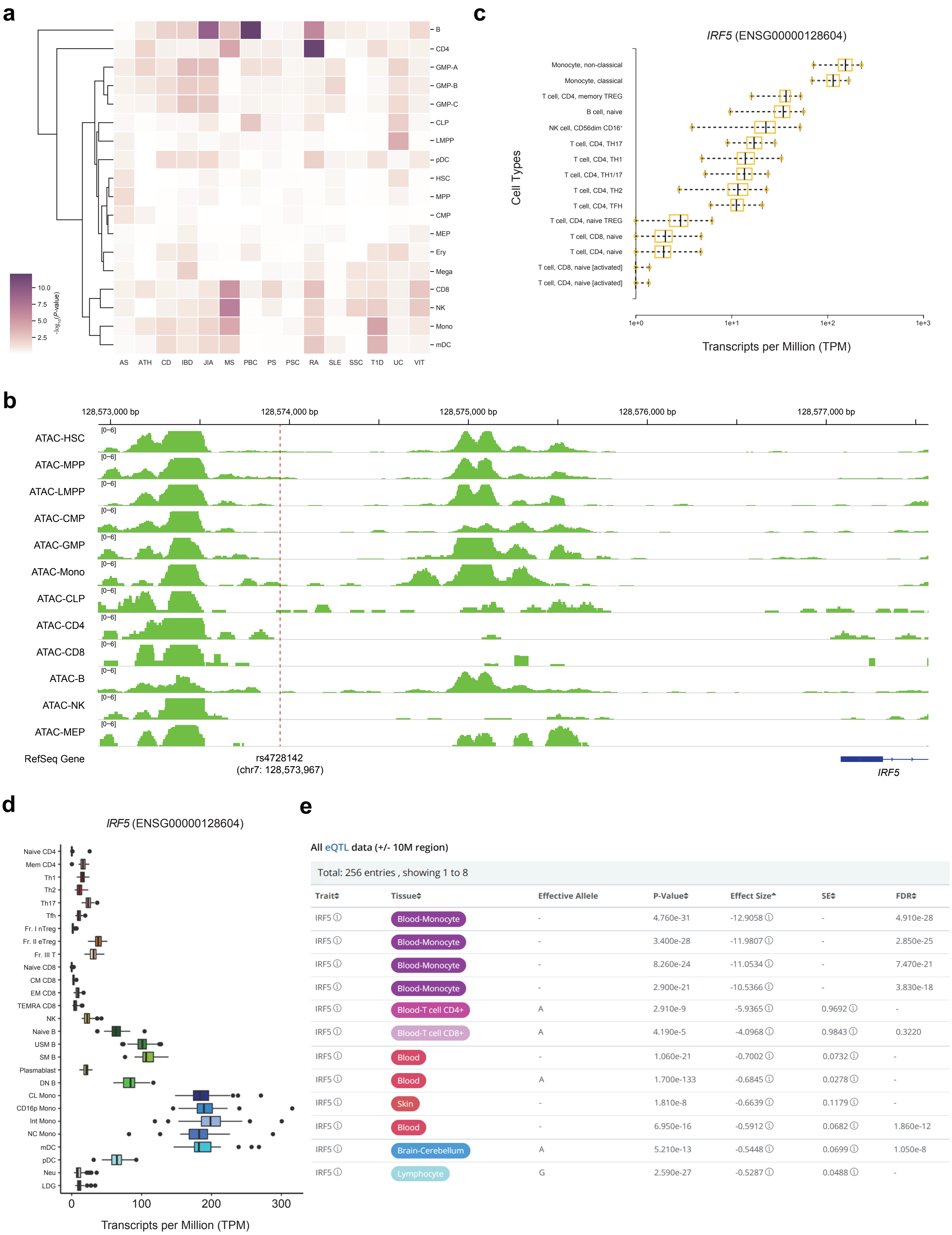

Trait	Tissue	Effective Allele	P-Value	Effect Size	SE	FDR
IRF5	Blood-Monocyte	-	4.760e-31	-12.9058	-	4.910e-28
IRF5	Blood-Monocyte	-	3.400e-28	-11.9807	-	2.850e-25
IRF5	Blood-Monocyte	-	8.260e-24	-11.0534	-	7.470e-21
IRF5	Blood-Monocyte	-	2.900e-21	-10.5366	-	3.830e-18
IRF5	Blood-T cell CD4+	A	2.910e-9	-5.9365	0.9692	-
IRF5	Blood-T cell CD8+	A	4.190e-5	-4.0968	0.9843	0.3220
IRF5	Blood	-	1.060e-21	-0.7002	0.0732	-
IRF5	Blood	A	1.700e-133	-0.6845	0.0278	-
IRF5	Skin	-	1.810e-8	-0.6639	0.1179	-
IRF5	Blood	-	6.950e-16	-0.5912	0.0682	1.860e-12
IRF5	Brain-Cerebellum	A	5.210e-13	-0.5448	0.0699	1.050e-8
IRF5	Lymphocyte	G	2.590e-27	-0.5287	0.0488	-

Supplementary Fig. 2 | The rs4728142-containing region shows the hematopoietic lineage-specific effect through chromatin state and gene expression analysis. a gchromVAR causal immune cell types estimation for different autoimmune diseases based on the GWAS summary statistics. Abbreviations for autoimmune diseases and immune cells: ankylosing spondylitis (AS), asthma (ATH), Crohn's disease (CD), inflammatory bowel disease (IBD), juvenile idiopathic arthritis (JIA), multiple sclerosis (MS), primary biliary cirrhosis (PBC), psoriasis (PS), primary sclerosing cholangitis (PSC), rheumatoid arthritis (RA), systemic lupus erythematosus (SLE), systemic sclerosis (SSC), type 1 diabetes (T1D), ulcerative colitis (UC), vitiligo (VIT). Abbreviations for hematopoietic cell types: B cell (B), $CD4^+$ T cell (CD4), granulocyte-macrophage progenitor cell (GMP), common lymphoid progenitor cell (CLP), lymphoid-primed multipotent progenitor cell (LMPP), plasmacytoid dendritic cell (pDC), hematopoietic stem cell (HSC), multipotent progenitor cell (MPP), common myeloid progenitor cell (CMP), megakaryocyte-erythroid progenitor cell (MEP), erythroid cell (Ery), megakaryocyte (Mega), $CD8^+$ T cell (CD8), natural killer cell (NK), monocyte (Mono), and myeloid dendritic cell (mDC). **b** Chromatin accessibility (ATAC-seq) of 12 normal hematopoietic cell types at the *IRF5* nearby region. Abbreviations for immune cells: hematopoietic stem cell (HSC), multipotent progenitor cell (MPP), lymphoid-primed multipotent progenitor cell (LMPP), common myeloid progenitor cell (CMP), granulocyte-macrophage progenitor cell (GMP), monocyte (Mono), common lymphoid progenitor cell (CLP), $CD4^+$ T cell (CD4), $CD8^+$ T cell (CD8), B cell (B), natural killer cell (NK), and megakaryocyte-erythroid progenitor cell (MEP). **c** *IRF5* expression data among different individuals across 15 immune cells in the DICE dataset. Box and whisker plot; boxes depict the upper and lower quartiles of the data, and whiskers depict the range of the data. **d** *IRF5* expression in the ImmuNexUT project, including 28 distinct immune cell subsets from 337 patients diagnosed with 10 categories of immune-mediated diseases and 79 healthy volunteers. **e** Effect size comparison of eQTL (rs4728142-*IRF5*) among different tissue/cell types in QTLbase.

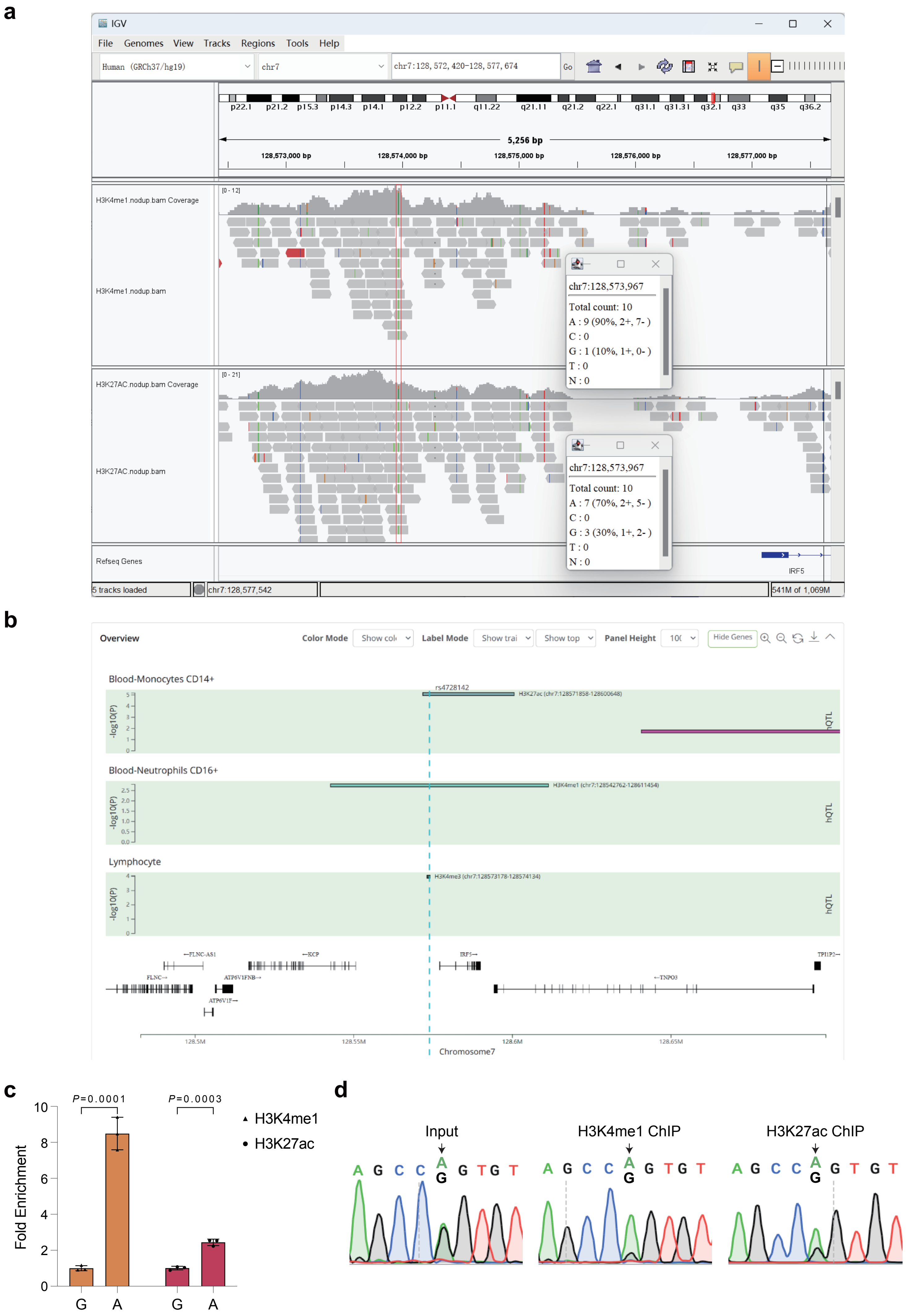

a
IGV
File Genomes View Tracks Regions Tools Help
Human (GRCh37/hg19)
chr7
chr7:128,572,420-128,577,674
Go
p22.1 p21.2 p15.3 p14.3 p14.1 p12.2 p11.1 q11.22 q21.11 q21.2 q22.1 q31.1 q31.31 q32.1 q33 q35 q36.2
5,256 bp
128,573,000 bp
128,574,000 bp
128,575,000 bp
128,576,000 bp
128,577,000 bp
H3K4me1.nodup.bam Coverage
H3K4me1.nodup.bam
[0 - 12]
chr7:128,573,967
Total count: 10
A : 9 (90%, 2+, 7-)
C : 0
G : 1 (10%, 1+, 0-)
T : 0
N : 0
H3K27AC.nodup.bam Coverage
H3K27AC.nodup.bam
[0 - 21]
chr7:128,573,967
Total count: 10
A : 7 (70%, 2+, 5-)
C : 0
G : 3 (30%, 1+, 2-)
T : 0
N : 0
Refseq Genes
IRF5
5 tracks loaded
chr7:128,577,542
541M of 1,069M
b
Overview
Color Mode
Label Mode
Show top
Panel Height
Hide Genes
Blood-Monocytes CD14+
rs4728142
H3K27ac (chr7:128571858-128600648)
-log10(P)
hQTL
Blood-Neutrophils CD16+
H3K4me1 (chr7:128542762-128611454)
Lymphocyte
H3K4me3 (chr7:128573178-128574134)
←FLNC-AS1
←KCP
IRF5→
TPI1P2→
FLNC→
ATP6V1FNB→
←TNPO3
ATP6V1F→
128.5M
128.55M
128.6M
128.65M
Chromosome7
c
Fold Enrichment
P=0.0001
P=0.0003
▲ H3K4me1
● H3K27ac
G
A
G
A
d
Input
H3K4me1 ChIP
H3K27ac ChIP
A G C C A G G T G T

Supplementary Fig. 3 | rs4728142-containing region shows a likely allele-specific effect on enhancer activity. a Allelic imbalance analysis of rs4728142 based on H3K4me1 and H3K27ac chromatin immunoprecipitation followed by sequencing (ChIP-seq) data of SC cells with heterozygous genotype. For H3K4me1, rs4728142 allele A receives 90% read coverage than 10% in allele G. For H3K27ac, rs4728142 allele A receives 70% read coverage than 30% in allele G. **b** Histone modification QTLs evidence of rs4728142 among different blood cell types in QTLbase. rs4728142 is documented as a H3K27ac QTL in Monocytes, a H3K4me1 QTL in Neutrophils, and a H3K4me3 QTL in Lymphocytes. **c** ChIP enrichments of H3K4me1 and H3K27ac at the rs4728142-containing region in SC cells as determined by allele-specific ChIP-qPCR. **d** Sanger sequencing chromatograms of H3K4me1 and H3K27ac ChIP-qPCR at the rs4728142 locus. Data are represented as the means ± SD, n = 3 biologically independent samples, and unpaired two-tailed Student's *t*-test is used to calculate *P*-values in **c**. Source data are provided as a Source Data file.

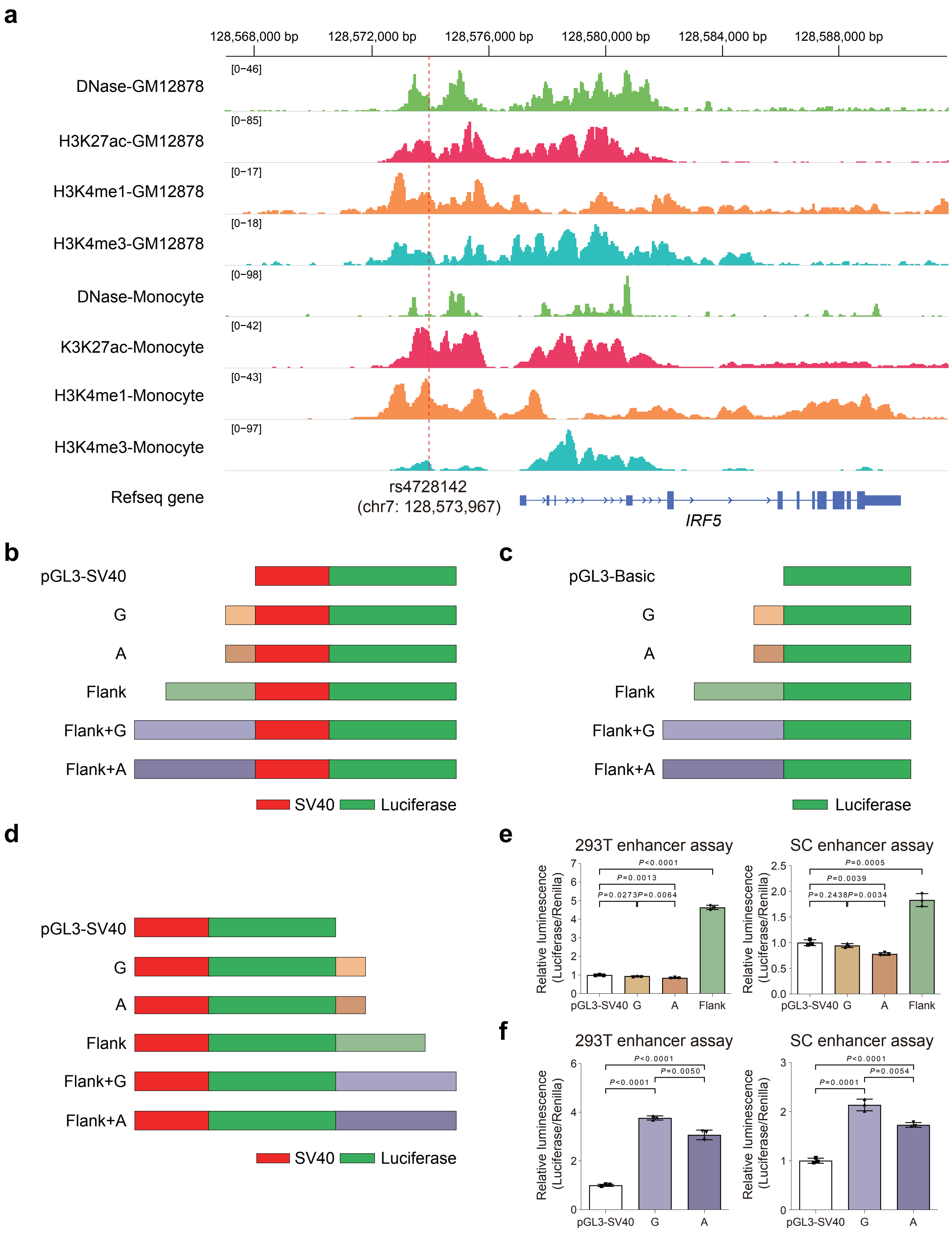

a
128,568,000 bp
128,572,000 bp
128,576,000 bp
128,580,000 bp
128,584,000 bp
128,588,000 bp
DNase-GM12878 [0−46]
H3K27ac-GM12878 [0−85]
H3K4me1-GM12878 [0−17]
H3K4me3-GM12878 [0−18]
DNase-Monocyte [0−98]
K3K27ac-Monocyte [0−42]
H3K4me1-Monocyte [0−43]
H3K4me3-Monocyte [0−97]
Refseq gene
rs4728142
(chr7: 128,573,967)
IRF5
b
pGL3-SV40
G
A
Flank
Flank+G
Flank+A
SV40
Luciferase
c
pGL3-Basic
G
A
Flank
Flank+G
Flank+A
Luciferase
d
pGL3-SV40
G
A
Flank
Flank+G
Flank+A
SV40
Luciferase
e
293T enhancer assay
SC enhancer assay
Relative luminescence (Luciferase/Renilla)
P<0.0001
P=0.0013
P=0.0273
P=0.0064
P=0.0005
P=0.0039
P=0.2438
P=0.0034
pGL3-SV40
G
A
Flank
f
293T enhancer assay
SC enhancer assay
Relative luminescence (Luciferase/Renilla)
P<0.0001
P=0.0050
P<0.0001
P=0.0054
P=0.0001
pGL3-SV40
G
A

Supplementary Fig. 4 | rs4728142 affects the activity of a promoter-like enhancer adjacent to *IRF5* promoter in monocytes. a Open chromatin (DNase-seq) and histone modification characteristics (H3K27ac, H3K4me1, and H3K4me3) at the *IRF5* nearby region in GM12878 lymphoblastoid cell line and $CD14^{+}$ monocytes from the Roadmap Epigenomics Project. **b**–**d** Strategies of luciferase reporter assays. All types of sequences were cloned into upstream of SV40 promoter in the pGL3-Promoter vector for enhancer activity detection (**b**), or upstream of luciferase reporter gene in the pGL3-Basic vector for promoter activity detection (**c**), or downstream of luciferase reporter gene in the pGL3-Promoter vector for enhancer activity detection (**d**). **e**, **f** Luciferase reporter assays utilizing vectors harboring the rs4728142-containing sequences with different alleles (the non-risk allele G or the risk allele A), or the upstream regulatory element (Termed Flank) (**e**), or the integrated sequences harboring the upstream enhancer and rs4728142-containing region with different alleles (G or A) (**f**) for enhancer assay in 293T and SC cells. Data are represented as the means ± SD, n = 3 biologically independent samples, and unpaired two-tailed Student's *t*-test is used to calculate *P*-values in **e** and **f**. Source data are provided as a Source Data file.

a

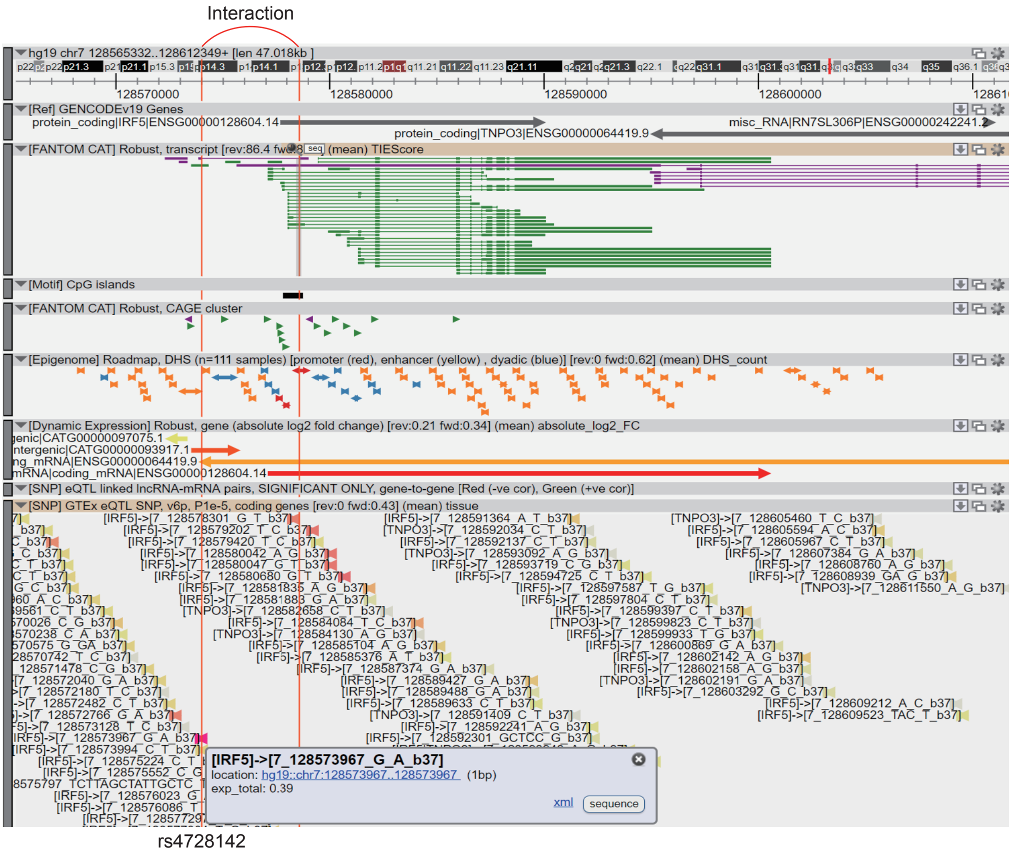

b

rs4728142 viewpoint (GRCh37/hg19 chr7: 128,573,714–128,574,338)

GATCCAAGGCCTGGAGGACAGGGTGGGGCCAGAACAGATTCCACCGACAAGGGCAGGAGCCAGGTTGCAGGCATCAGAGAG
GACAGGTGACCTGTCGGGGCCTGGAGCAAGGGCCCGGGCAGCTTCCTCCACAGGCCATGTGAAGACAGAGCCATGGATTGG
GAAAGGTGGAGACTCCGAGTGTAGAGGTGTCCATGTAACAGTGCTGGCTGAATGGGGGACTTCCAGGTCACACCCCAAAAAGC
TCTGAGCCAGTGTTAGTAAGAAATGGGGAGGAAGGCCCAGCTGTGGCGCCCTGGGCATTAATCCTAAAAAAAAAGATACTACTT
CTATGTGCCAGAAACTTCAAAGCAAGTGAACACTTCAAGTACGAGAACCAGAGGTGTTTTTTTGTTTTTTCTTTTTTCTTTTGAGA
CGGAGTATCGCTCTGTCACCCAGGCTGGAGTGCAATGGTGCAATCTCGGCTCACTACAACCTCCGCCTTCTGGGTTCAAGCAAT
TTTCCTGCCTCAGCTTCCCGAGTAGCTGGGATTACAAGTATGAACCACCACGCCTGGCTAACTTTTGTATTTTTTTTTTTTTTAGTA
GAGACGGGGTTTCCCCATGTTGGCCAGGCTGGTCTCGATC

The region (GRCh37/hg19 chr7: 128,578,363–128,578,537) in *IRF5* downstream alternative promoter with the highest interaction frequency

GATCGGTTTGGGGTGCTGGCGCCCGGGGAGCCCAGTGACCCAGGCGGCGGAGTGGGCAGCGCTGCGGGGGGCGCCGGCTC
TGCTGCTCTCCCTCCCCCTCGCCATCGCCCAGAATGGGGGTTCCCGGGAGCCCCGCTGGAGGCTGGCTTGGACCACAGAGGA
GCGAGGCCCGATC

c

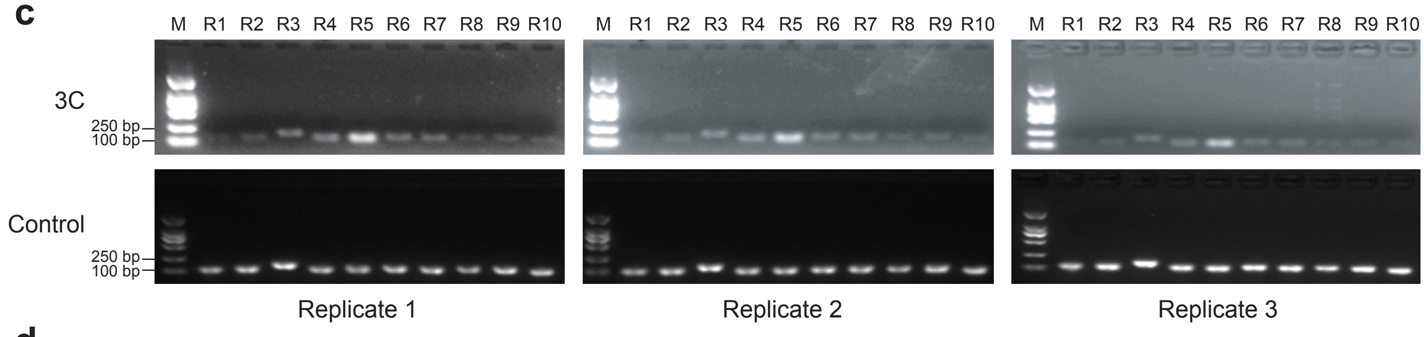

d

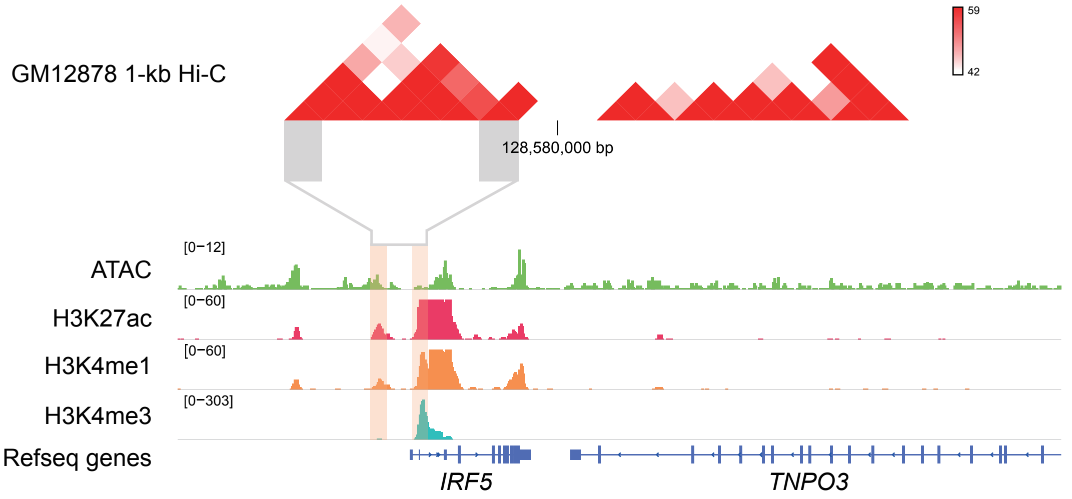

Supplementary Fig. 5 | The rs4728142-containing region interacts with the *IRF5* downstream alternative promoter in an allele-specific manner. a *IRF5* promoter signature and transcript information retrieved from the FANTOM CAT Browser. Position information of the rs4728142-containing 3C/4C viewpoint and the sequence in the *IRF5* downstream alternative promoter with the highest interaction frequency is marked. **b** Sequence information for the rs4728142-containing region 3C/4C viewpoint and the sequence in the *IRF5* downstream alternative promoter with the highest interaction frequency. Sequences with yellow background represent the putative ZBTB3 binding motif as analyzed by CIS-BP and FIMO. Red letter represents the rs4728142 risk variant, and underlining letters represent the used restriction sites. **c** Electrophoresis results of 3C assay for the rs4728142-containing region in SC cells. The samples derive from the same experiment and that gels were processed in parallel. **d** 1-kb contact map visualization around the rs4728142-containing region and the *IRF5* alternative promoters using the GM12878 Hi-C data.

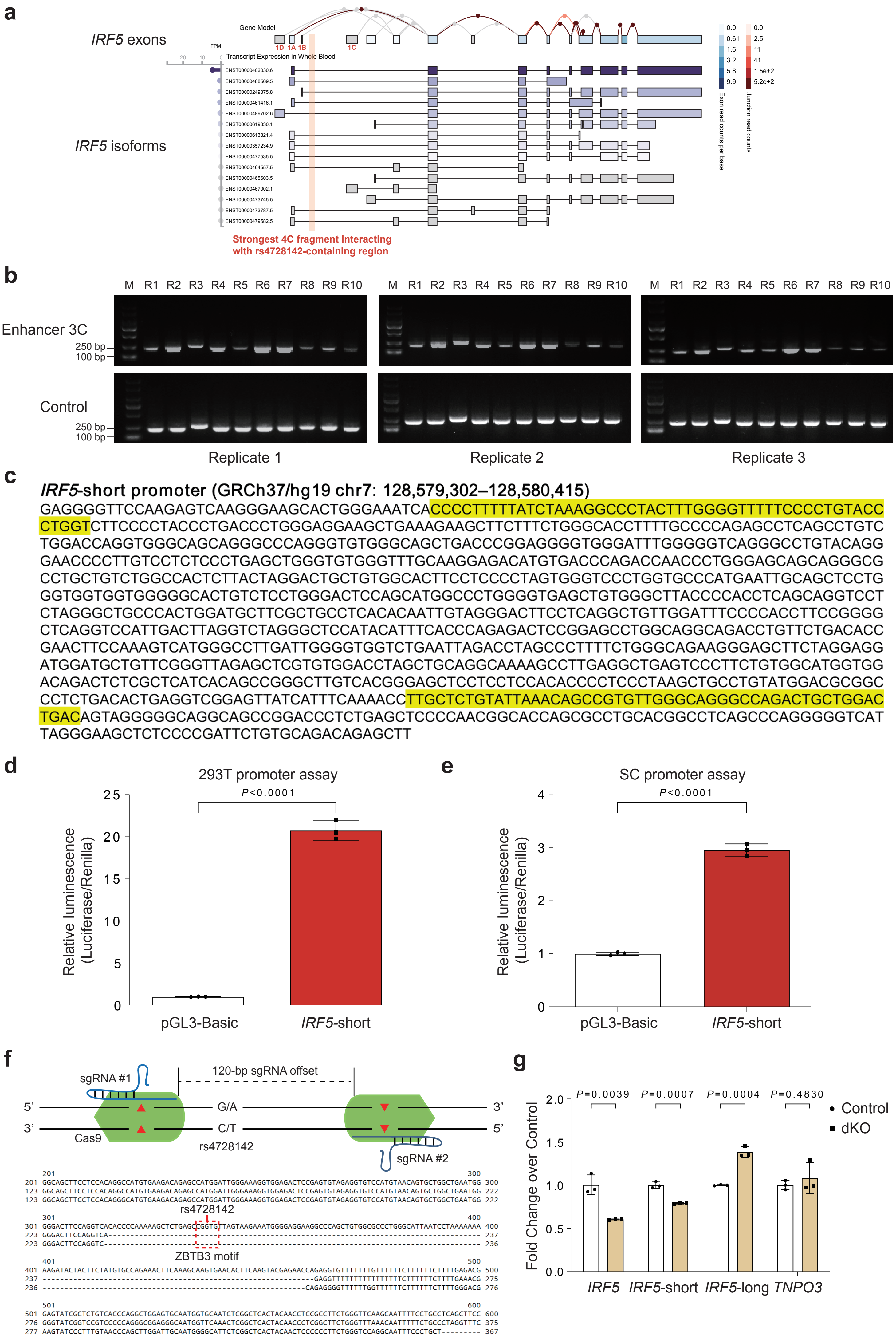

a
IRF5 exons
IRF5 isoforms
Gene Model
Transcript Expression in Whole Blood
TPM
1D 1A 1B
1C
Exon read counts per base
Junction read counts
Strongest 4C fragment interacting with rs4728142-containing region
b
M R1 R2 R3 R4 R5 R6 R7 R8 R9 R10
Enhancer 3C
Control
250 bp
100 bp
Replicate 1
Replicate 2
Replicate 3
c
IRF5-short promoter (GRCh37/hg19 chr7: 128,579,302–128,580,415)
GAGGGGTTCCAAGAGTCAAGGGAAGCACTGGGAAATCACCCCTTTTTATCTAAAGGCCCTACTTTGGGGTTTTTCCCCTGTACC
CTGGTCTTCCCCTACCCTGACCCTGGGAGGAAGCTGAAAGAAGCTTCTTTCTGGGCACCTTTTGCCCCAGAGCCTCAGCCTGTC
TGGACCAGGTGGGCAGCAGGGCCCAGGGTGTGGGCAGCTGACCCGGAGGGGTGGGATTTGGGGGTCAGGGCCTGTACAGG
GAACCCCTTGTCCTCTCCCTGAGCTGGGTGTGGGTTTGCAAGGAGACATGTGACCCAGACCAACCCTGGGAGCAGCAGGGCG
CCTGCTGTCTGGCCACTCTTACTAGGACTGCTGTGGCACTTCCTCCCCTAGTGGGTCCCTGGTGCCCATGAATTGCAGCTCCTG
GGTGGTGGTGGGGGCACTGTCTCCTGGGACTCCAGCATGGCCCTGGGGTGAGCTGTGGGCTTACCCCACCTCAGCAGGTCCT
CTAGGGCTGCCCACTGGATGCTTCGCTGCCTCACACAATTGTAGGGACTTCCTCAGGCTGTTGGATTTCCCCACCTTCCGGGG
CTCAGGTCCATTGACTTAGGTCTAGGGCTCCATACATTTCACCCAGAGACTCCGGAGCCTGGCAGGCAGACCTGTTCTGACACC
GAACTTCCAAAGTCATGGGCCTTGATTGGGGTGGTCTGAATTAGACCTAGCCCTTTTCTGGGCAGAAGGGAGCTTCTAGGAGG
ATGGATGCTGTTCGGGTTAGAGCTCGTGTGGACCTAGCTGCAGGCAAAAGCCTTGAGGCTGAGTCCCTTCTGTGGCATGGTGG
ACAGACTCTCGCTCATCACAGCCGGGCTTGTCACGGGAGCTCCTCCTCCACACCCCTCCCTAAGCTGCCTGTATGGACGCGGC
CCTCTGACACTGAGGTCGGAGTTATCATTTCAAAACCTTGCTCTGTATTAAACAGCCGTGTTGGGCAGGGCCAGACTGCTGGAC
TGACAGTAGGGGGCAGGCAGCCGGACCCTCTGAGCTCCCCAACGGCACCAGCGCCTGCACGGCCTCAGCCCAGGGGGTCAT
TAGGGAAGCTCTCCCCGATTCTGTGCAGACAGAGCTT
d
293T promoter assay
P<0.0001
Relative luminescence (Luciferase/Renilla)
pGL3-Basic
IRF5-short
e
SC promoter assay
P<0.0001
Relative luminescence (Luciferase/Renilla)
pGL3-Basic
IRF5-short
f
sgRNA #1
120-bp sgRNA offset
5'
3'
G/A
C/T
Cas9
rs4728142
sgRNA #2
ZBTB3 motif
g
P=0.0039
P=0.0007
P=0.0004
P=0.4830
Control
dKO
Fold Change over Control
IRF5
IRF5-short
IRF5-long
TNPO3

Supplementary Fig. 6 | The rs4728142-containing region orchestrates chromatin looping to regulate *IRF5* alternative promoter usage. a *IRF5* transcript diagram retrieved from the GTEx Portal Transcript Browser. Four *IRF5* alternative promoters (including transcription start sites [TSSs] from exon 1D, 1A, 1B, and 1C) are highlighted, and the chromatin interaction region with the highest looping intensity separates the *IRF5* alternative promoters into upstream (TSSs for exon 1D, 1A, and 1B) and downstream promoters (TSSs for exon 1C). **b** Electrophoresis results of 3C assay for the rs4728142-associated enhancer in SC cells. The samples derive from the same experiment and that gels were processed in parallel. **c** Sequence information of the putative *IRF5*-short promoter analyzed by the BDGP Browser. Sequences with yellow background represent the predicted promoter core regions. **d**, **e** Promoter luciferase assays for the putative *IRF5*-short promoter in 293T (**d**) and SC (**e**) cells. **f** Strategy of rs4728142 double-knockout (dKO, top) and Sanger sequencing result as analyzed by CRISP-ID Web Portal (bottom). sgRNA offset between two sgRNAs is 120-bp. Red triangle denotes the cutting site, red arrow denotes the rs4728142 location, and red dotted box denotes the ZBTB3 core motif sequence. **g** *IRF5* transcript expression in the rs4728142-dKO SC-Cas9 cells compared with the unedited SC-Cas9 cells (control) as determined by RT-qPCR. *TNPO3* is the gene adjacent to the upstream *IRF5* gene. Data are represented as the means ± SD, n = 3 biologically independent samples, and unpaired two-tailed Student's *t*-test is used to calculate *P*-values in **d**, **e** and **g**. Source data are provided as a Source Data file.

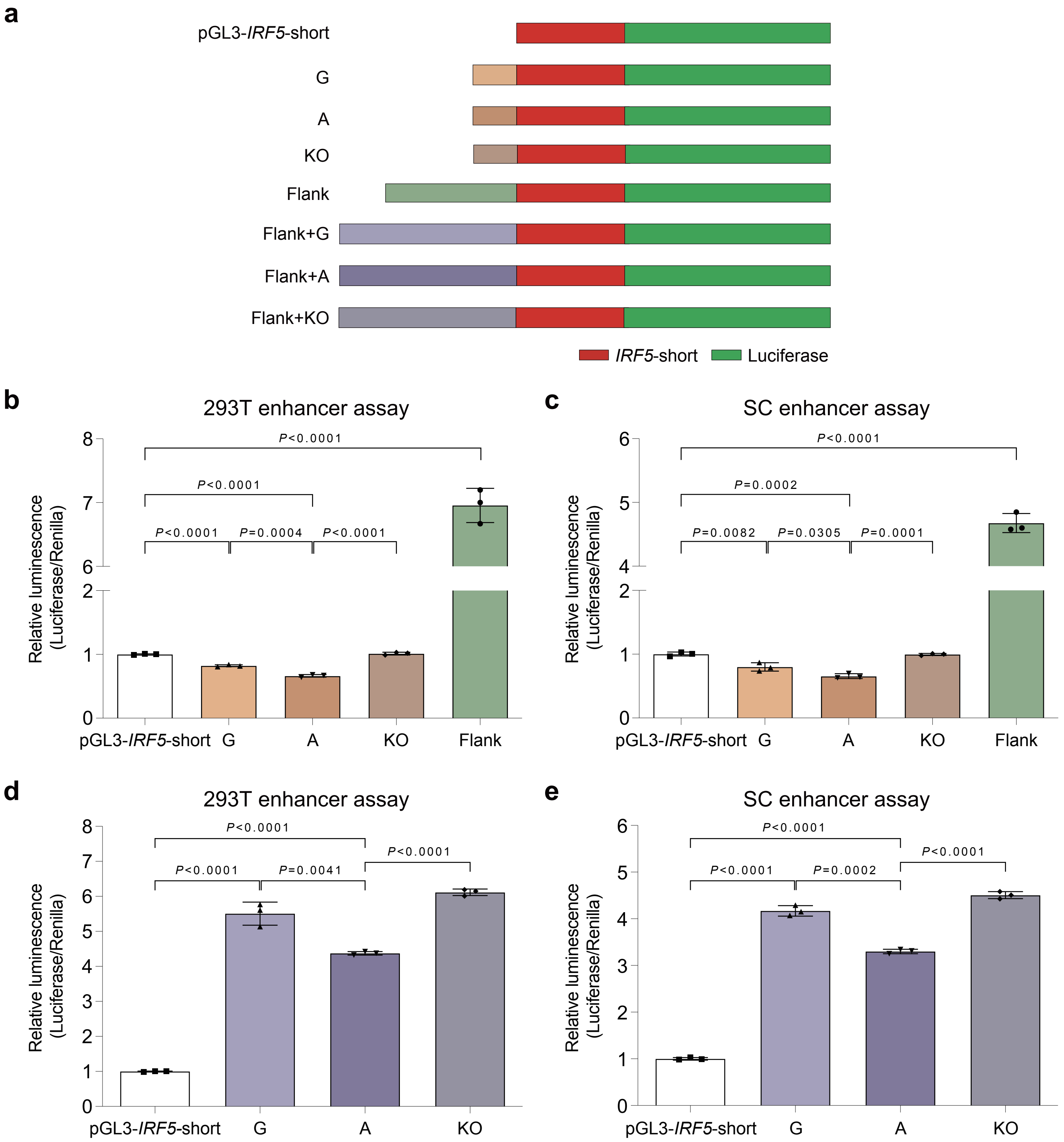

a
pGL3-IRF5-short
G
A
KO
Flank
Flank+G
Flank+A
Flank+KO
IRF5-short
Luciferase
b
293T enhancer assay
Relative luminescence (Luciferase/Renilla)
P<0.0001
P<0.0001
P<0.0001
P=0.0004
P<0.0001
pGL3-IRF5-short
G
A
KO
Flank
c
SC enhancer assay
Relative luminescence (Luciferase/Renilla)
P<0.0001
P=0.0002
P=0.0082
P=0.0305
P=0.0001
pGL3-IRF5-short
G
A
KO
Flank
d
293T enhancer assay
Relative luminescence (Luciferase/Renilla)
P<0.0001
P<0.0001
P<0.0001
P=0.0041
pGL3-IRF5-short
G
A
KO
e
SC enhancer assay
Relative luminescence (Luciferase/Renilla)
P<0.0001
P<0.0001
P<0.0001
P=0.0002
pGL3-IRF5-short
G
A
KO

Supplementary Fig. 7 | rs4728142 affects the activity of a promoter-like enhancer adjacent to *IRF5* promoter. a Strategies of luciferase reporter assays. All types of sequences were cloned into upstream of *IRF5*-short promoter in the pGL3-*IRF5*-short vector for enhancer activity detection. **b**, **c** Luciferase reporter assays utilizing vectors harboring the rs4728142-containing sequences with different alleles (G or A) or rs4728142 allele knockout (KO), or the upstream regulatory element (Termed Flank) for enhancer assay in 293T (**b**) and SC (**c**) cells. **d**, **e** Luciferase reporter assays utilizing vectors harboring the integrated sequences harboring the upstream regulatory element and rs4728142-containing sequence with different alleles (G or A) or rs4728142-KO for enhancer assay in 293T (**d**) and SC (**e**) cells. Data are represented as the means ± SD, n = 3 biologically independent samples, and unpaired two-tailed Student's *t*-test is used to calculate *P*-values in **b**–**e**. Source data are provided as a Source Data file.

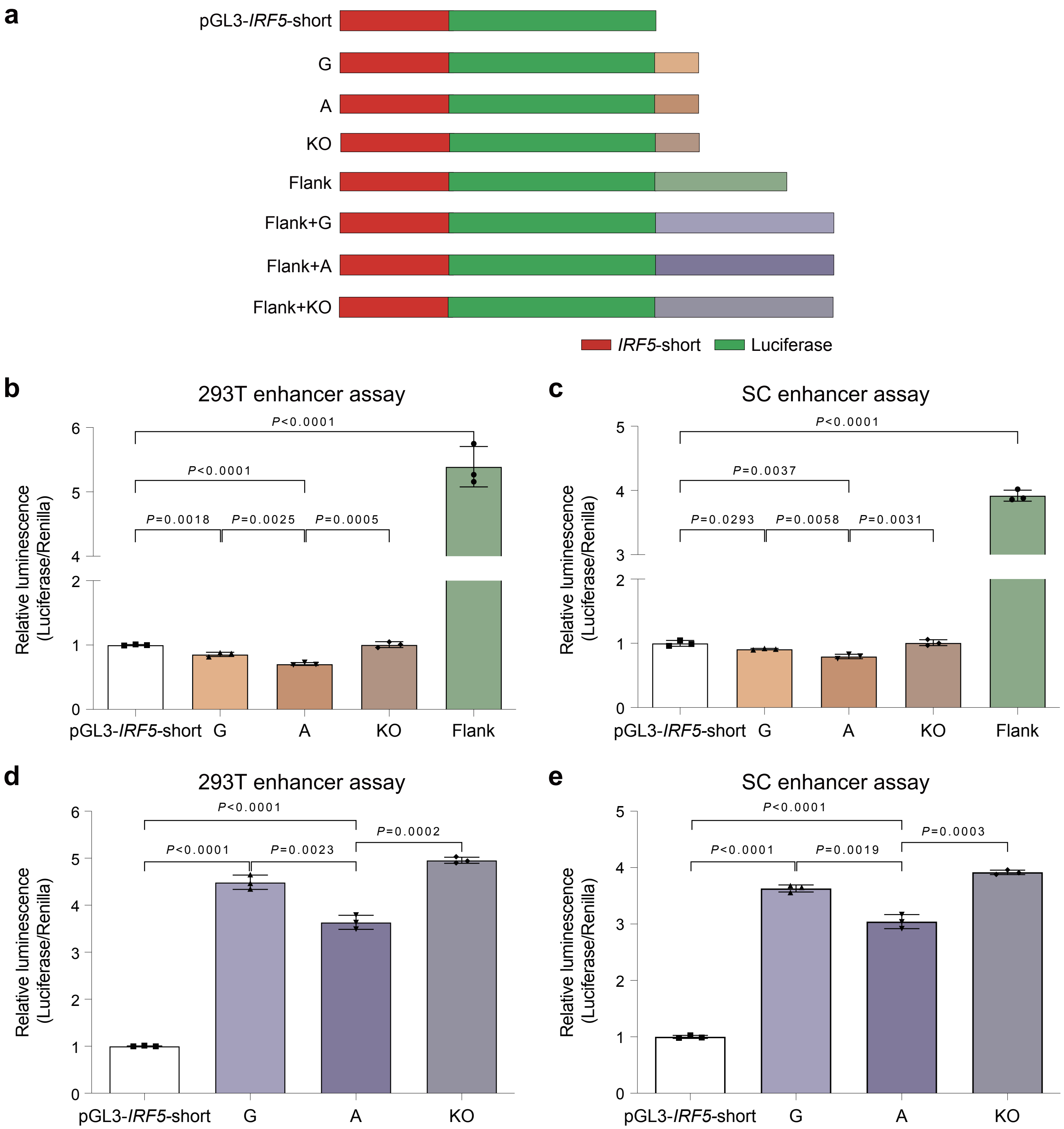
a
pGL3-IRF5-short
G
A
KO
Flank
Flank+G
Flank+A
Flank+KO
IRF5-short
Luciferase
b
293T enhancer assay
Relative luminescence (Luciferase/Renilla)
P<0.0001
P<0.0001
P=0.0018
P=0.0025
P=0.0005
pGL3-IRF5-short
G
A
KO
Flank
c
SC enhancer assay
Relative luminescence (Luciferase/Renilla)
P<0.0001
P=0.0037
P=0.0293
P=0.0058
P=0.0031
pGL3-IRF5-short
G
A
KO
Flank
d
293T enhancer assay
Relative luminescence (Luciferase/Renilla)
P<0.0001
P=0.0002
P<0.0001
P=0.0023
pGL3-IRF5-short
G
A
KO
e
SC enhancer assay
Relative luminescence (Luciferase/Renilla)
P<0.0001
P=0.0003
P<0.0001
P=0.0019
pGL3-IRF5-short
G
A
KO

Supplementary Fig. 8 | s4728142 affects the activity of a promoter-like enhancer adjacent to *IRF5* promoter. a Strategies of luciferase reporter assays. All types of sequences were cloned into downstream of luciferase reporter gene in the pGL3-*IRF5*-short vector for enhancer activity detection. **b**, **c** Luciferase reporter assays utilizing vectors harboring the rs4728142-containing sequences with different alleles (G or A) or rs4728142-KO, or the upstream regulatory element (Termed Flank) for enhancer assay in 293T (**b**) and SC (**c**) cells. **d**, **e** Luciferase reporter assays utilizing vectors harboring the integrated sequences harboring the upstream regulatory element and rs4728142-containing sequence with different alleles (G or A) or rs4728142-KO for enhancer assay in 293T (**d**) and SC (**e**) cells. Data are represented as the means ± SD, n = 3 biologically independent samples, and unpaired two-tailed Student's *t*-test is used to calculate *P*-values in **b**–**e**. Source data are provided as a Source Data file.

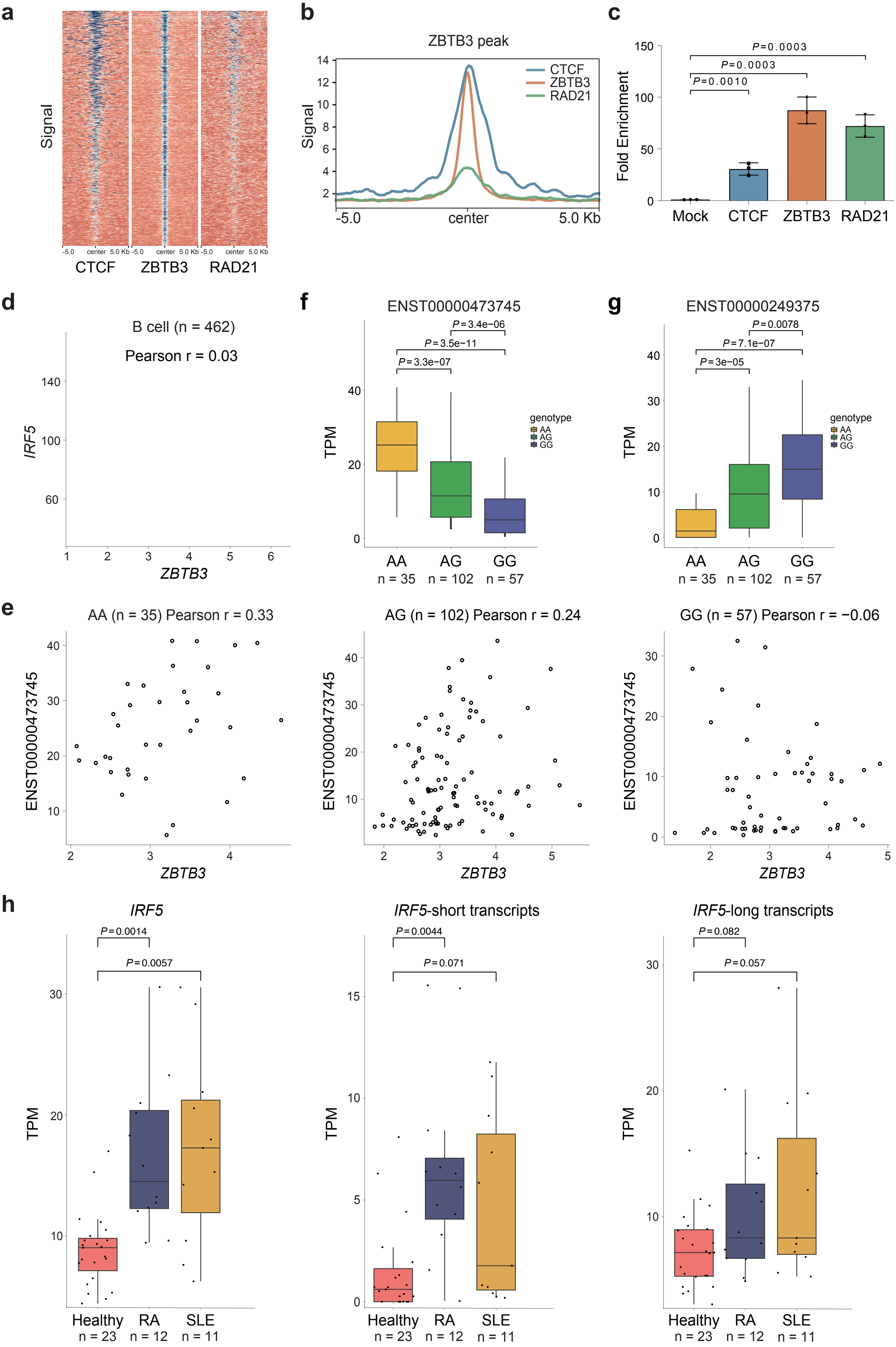

a
Signal
CTCF
ZBTB3
RAD21
b
ZBTB3 peak
Signal
CTCF
ZBTB3
RAD21
-5.0
center
5.0 Kb
c
Fold Enrichment
P = 0.0003
P = 0.0003
P = 0.0010
Mock
CTCF
ZBTB3
RAD21
d
B cell (n = 462)
Pearson r = 0.03
IRF5
ZBTB3
f
ENST00000473745
P = 3.4e−06
P = 3.5e−11
P = 3.3e−07
TPM
genotype
AA
AG
GG
n = 35
n = 102
n = 57
g
ENST00000249375
P = 0.0078
P = 7.1e−07
P = 3e−05
e
AA (n = 35) Pearson r = 0.33
AG (n = 102) Pearson r = 0.24
GG (n = 57) Pearson r = −0.06
ENST00000473745
ZBTB3
h
IRF5
P = 0.0014
P = 0.0057
IRF5-short transcripts
P = 0.0044
P = 0.071
IRF5-long transcripts
P = 0.082
P = 0.057
Healthy
RA
SLE
n = 23
n = 12
n = 11

Supplementary Fig. 9 | ZBTB3 mediates the allele-specific chromatin looping to regulate *IRF5*-short transcript expression at the rs4728142 locus. a Heatmaps of CTCF, ZBTB3, and RAD21 ChIP-seq signals surrounding ± 5-kb of ZBTB3 binding peaks. **b** Average profiles of CTCF, ZBTB3, and RAD21 across a genomic window of ± 5-kb surrounding ZBTB3 binding peaks. **c** ChIP enrichments of CTCF, ZBTB3, and RAD21 at the rs4728142 locus as determined by ChIP-qPCR. **d** Correlation analysis of *ZBTB3* and *IRF5* expression in 462 human GM12878 lymphoblastoid B cell RNA-seq samples from the Geuvadis dataset. **e** Correlation analysis of *ZBTB3* and the highest expressed *IRF5*-short transcript (ENST00000473745) in 194 $CD14^+$ monocyte RNA-seq samples (AA n = 35, AG n = 102, GG n = 57) from the BLUEPRINT dataset. **f**, **g** Transcript-level eQTL analysis for correlation between the rs4728142 genotype and the highest expressed *IRF5*-short transcript (ENST00000473745), Box and whisker plot; boxes depict the upper and lower quartiles of the data, and whiskers depict the range of the data. (**f**) or *IRF5*-long transcript (ENST00000249375) (**g**) using 194 monocyte RNA-seq samples from the BLUEPRINT dataset. **h** Comparison of *IRF5* gene, *IRF5*-short, and *IRF5*-long transcripts expression in blood RNA-seq samples between healthy individuals and RA/SLE patients (healthy n = 23, RA n = 12, SLE n = 11, Mann Whitney U Test). Box and whisker plot; boxes depict the upper and lower quartiles of the data, and whiskers depict the range of the data. Data are represented as the means ± SD, n = 3 biologically independent samples, and unpaired two-tailed Student's *t*-test is used to calculate *P*-values in **c**. Source data are provided as a Source Data file.

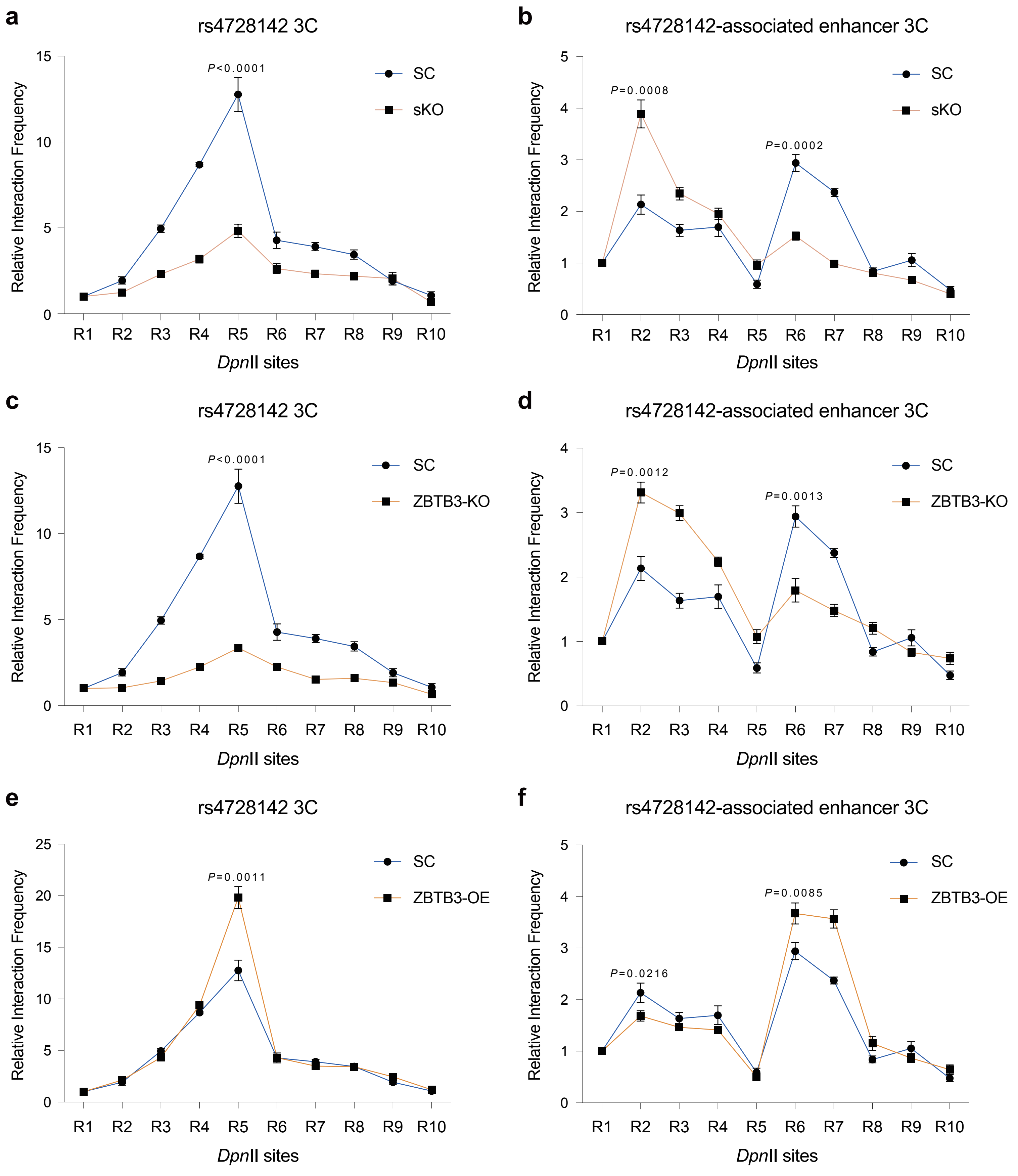
a
rs4728142 3C
P<0.0001
SC
sKO
Relative Interaction Frequency
0
5
10
15
R1 R2 R3 R4 R5 R6 R7 R8 R9 R10
DpnII sites
b
rs4728142-associated enhancer 3C
P=0.0008
P=0.0002
SC
sKO
Relative Interaction Frequency
0
1
2
3
4
5
R1 R2 R3 R4 R5 R6 R7 R8 R9 R10
DpnII sites
c
rs4728142 3C
P<0.0001
SC
ZBTB3-KO
Relative Interaction Frequency
0
5
10
15
R1 R2 R3 R4 R5 R6 R7 R8 R9 R10
DpnII sites
d
rs4728142-associated enhancer 3C
P=0.0012
P=0.0013
SC
ZBTB3-KO
Relative Interaction Frequency
0
1
2
3
4
R1 R2 R3 R4 R5 R6 R7 R8 R9 R10
DpnII sites
e
rs4728142 3C
P=0.0011
SC
ZBTB3-OE
Relative Interaction Frequency
0
5
10
15
20
25
R1 R2 R3 R4 R5 R6 R7 R8 R9 R10
DpnII sites
f
rs4728142-associated enhancer 3C
P=0.0216
P=0.0085
SC
ZBTB3-OE
Relative Interaction Frequency
0
1
2
3
4
5
R1 R2 R3 R4 R5 R6 R7 R8 R9 R10
DpnII sites

Supplementary Fig. 10 | ZBTB3 orchestrates chromatin looping to regulate *IRF5* alternative promoter usage by binding the rs4728142-containing region. a Relative interaction frequencies between the rs4728142-containing region and representative *Dpn*II cutting fragments indicated in Fig. 3b in SC and rs4728142 single-knockout (sKO) SC cells. **b** Relative interaction frequencies between the rs4728142-associated enhancer and representative *Dpn*II cutting fragments indicated in Fig. 4b in SC and rs4728142-sKO SC cells. **c** Relative interaction frequencies between the rs4728142-containing region and representative *Dpn*II cutting fragments indicated in Fig. 3b in SC and ZBTB3 knockout (KO) SC cells. **d** Relative interaction frequencies between the rs4728142-associated enhancer and representative *Dpn*II cutting fragments indicated in Fig. 4b in SC and ZBTB3-KO SC cells. **e** Relative interaction frequencies between the rs4728142-containing region and representative *Dpn*II cutting fragments indicated in Fig. 3b in SC and ZBTB3 overexpression (OE) SC cells. **f** Relative interaction frequencies between the rs4728142-associated enhancer and representative *Dpn*II cutting fragments indicated in Fig. 4b in SC and ZBTB3-OE SC cells. The samples derive from the same experiment and that gels were processed in parallel. Data are represented as the means ± SD, n = 3 biologically independent samples, and unpaired two-tailed Student's *t*-test is used to calculate *P*-values in **a**–**f**. Source data are provided as a Source Data file.

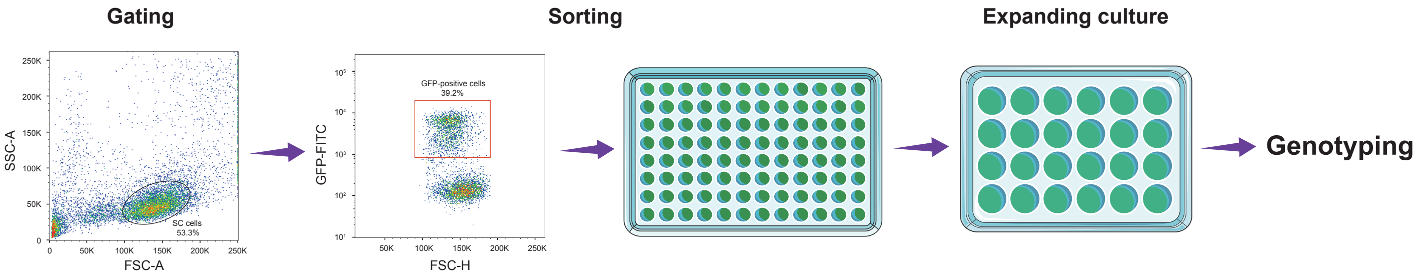
Gating
Sorting
Expanding culture
SSC-A
250K
200K
150K
100K
50K
0
50K
100K
150K
200K
250K
FSC-A
SC cells
53.3%
GFP-FITC
GFP-positive cells
39.2%
FSC-H
Genotyping

Supplementary Fig. 11 | Strategy of the flow cytometry gating and sorting in genome editing. Single SC cells were gated, and the single GFP-positive SC cells were sorted into the 96-well plates. After expending culture of the sorted cells from 96-well plates to 24-well plates, genomes of the cells were separately extracted for genotyping.
