# Supplementary material for: An autoimmune pleiotropic SNP modulates IRF5 alternative promoter usage through ZBTB3-mediated chromatin looping
Source: Nat Commun. 2023 Mar 3;14:1208. doi: 10.1038/s41467-023-36897-z (PMC9984425; doi:10.1038/s41467-023-36897-z)
Supplement: Supplementary file 3 — Description of Additional Supplementary Files [file 41467_2023_36897_MOESM3_ESM.pdf]

Description of Additional Supplementary Files

File Name: Supplementary Data 1
Description: Genome-wide studies for identification of the potential pleiotropic or shared genetic loci associated with autoimmune diseases.

File Name: Supplementary Data 2
Description: The curated sentinel pleiotropic variants associated with at least two autoimmune diseases.

File Name: Supplementary Data 3
Description: Study description of the collected full genome-wide association studies (GWAS) summary statistics of autoimmune diseases.

File Name: Supplementary Data 4
Description: gwas-pw variant-level pleiotropy estimation for the curated autoimmune pleiotropic variants. Summary statistics was derived from gwas-pw variant-level pleiotropy test.

File Name: Supplementary Data 5
Description: Study description of the collected fine-mappable GWAS summary statistics of autoimmune diseases.

File Name: Supplementary Data 6
Description: Likely causal variant estimation for each of the curated pleiotropic loci using public GWAS summary statistics of autoimmune diseases. Summary statistics was derived from fine-mapping algorithms.

File Name: Supplementary Data 7
Description: Functional annotation and prediction of the curated pleiotropic variants associated with multiple autoimmune diseases.

File Name: Supplementary Data 8
Description: Expression quantitative trait locus (eQTL) colocalization result (RTC) between the sentinel pleiotropic variants and five blood-derived whole-genome sequencing (WGS) eQTL datasets.

File Name: Supplementary Data 9
Description: Annotation information of the potential target genes of the pleiotropic variants associated with multiple autoimmune diseases.

File Name: Supplementary Data 10
Description: Evidence-based prioritization of the curated pleiotropic variant by considering reproducibility of pleiotropy, autoimmune disease causality, eQTL colocalization, and variant regulatory potential.

File Name: Supplementary Data 11
Description: rs4728142 is a transcript-Level eQTL associated with IRF5 promoter usage on macrophages exposed to inflammatory and metabolic stimulus. eQTL summary statistics was derived from transcript usage QTL (tuQTL) study (Kaur Alasoo et al. Elife. 2019 Jan 8;8:e41673).

File Name: Supplementary Data 12
Description: FIMO motif scanning result for the different alleles of rs4728142 surrounding sequence. Summary statistics was derived from FIMO motif scanning algorithm.

File Name: Supplementary Data 13
Description: GM12878 RAD21 chromatin interaction analysis by paired-end tag sequencing (ChIA-PET) analysis on the interaction between rs4728142 and the IRF5 downstream alternative promoter.

File Name: Supplementary Data 14
Description: Primers and oligonucleotides used in this study.
