# Supplementary material for: An autoimmune pleiotropic SNP modulates IRF5 alternative promoter usage through ZBTB3-mediated chromatin looping
Source: Nat Commun. 2023 Mar 3;14:1208. doi: 10.1038/s41467-023-36897-z (PMC9984425; doi:10.1038/s41467-023-36897-z)
Supplement: Supplementary file 18 — Reporting Summary [file 41467_2023_36897_MOESM18_ESM.pdf]

nature portfolio

Corresponding author(s): Mulin Jun Li

Last updated by author(s): Jan 6, 2023

Reporting Summary

Nature Portfolio wishes to improve the reproducibility of the work that we publish. This form provides structure for consistency and transparency in reporting. For further information on Nature Portfolio policies, see our Editorial Policies and the Editorial Policy Checklist.

Statistics

For all statistical analyses, confirm that the following items are present in the figure legend, table legend, main text, or Methods section.

n/a	Confirmed	
☐	☒	The exact sample size (*n*) for each experimental group/condition, given as a discrete number and unit of measurement
☐	☒	A statement on whether measurements were taken from distinct samples or whether the same sample was measured repeatedly
☐	☒	The statistical test(s) used AND whether they are one- or two-sided<br>*Only common tests should be described solely by name; describe more complex techniques in the Methods section.*
☐	☒	A description of all covariates tested
☐	☒	A description of any assumptions or corrections, such as tests of normality and adjustment for multiple comparisons
☐	☒	A full description of the statistical parameters including central tendency (e.g. means) or other basic estimates (e.g. regression coefficient) AND variation (e.g. standard deviation) or associated estimates of uncertainty (e.g. confidence intervals)
☐	☒	For null hypothesis testing, the test statistic (e.g. *F*, *t*, *r*) with confidence intervals, effect sizes, degrees of freedom and *P* value noted<br>*Give P values as exact values whenever suitable.*
☒	☐	For Bayesian analysis, information on the choice of priors and Markov chain Monte Carlo settings
☒	☐	For hierarchical and complex designs, identification of the appropriate level for tests and full reporting of outcomes
☐	☒	Estimates of effect sizes (e.g. Cohen's *d*, Pearson's *r*), indicating how they were calculated

Our web collection on statistics for biologists contains articles on many of the points above.

Software and code

Policy information about availability of computer code

Data collection

The data associated with the curated genome-wide studies which collected from PubMed and literature, are listed at Supplementary Data. Summary statistics of UKBB cohort were downloaded from Neale Lab UKBB (v3) (http://www.nealelab.is/uk-biobank), Gene ATLAS (Canela-Xandri et al., 2018), and GWAS ATLAS (Watanabe et al., 2019). Summary statistics of non-UKBB cohorts were retrieved from several public databases, including GWAS Catalog (Buniello et al., 2019) and ImmunoBase (https://genetics.opentargets.org/immunobase).

Data analysis

All software used for data analysis are publicly available.
R (v3.3.3) R Core Team, 2017 https://www.r-project.org/
RStudio (v1.0.136) RStudio Team, 2016 https://rstudio.com/
SnapGene Viewer (v4.3.10) SnapGene Software https://www.snapgene.com/
GraphPad Prism (v8.1.1) GraphPad Software https://www.graphpad.com/
Primer Premier 6.25 PREMIER Biosoft http://www.premierbiosoft.com/
CRISP-ID (v1.1) Dehairs et al., 2016 http://crispid.gbiomed.kuleuven.be/
CRISPOR (v4.99) Haeussler et al., 2016 http://crispor.tefor.net/
CHOPCHOP (v3) Labun et al., 2016 http://chopchop.cbu.uib.no/
UKBB (v3) UK BIOBANK http://www.nealelab.is/uk-biobank
Gene ATLAS Canela-Xandri et al., 2018 http://geneatlas.roslin.ed.ac.uk/
GWAS ATLAS (2019 release) Watanabe et al., 2019 https://bigd.big.ac.cn/gwas/
GWAS Catalog (v1.0.2) Buniello et al., 2019 https://www.ebi.ac.uk/gwas/
ImmunoBase Open Targets Genetics (19.05.05) https://genetics.opentargets.org/immunobase
GENCODE V28 Frankish et al., 2019 https://www.gencodegenes.org/
FANTOM CAT Hon et al., 2017 https://fantom.gsc.riken.jp/cat/

The 3D Genome Browser (2019 release) Wang et al., 2018 http://3dgenome.fsm.northwestern.edu/
DICE (2020 release) Schmiedel et al., 2018 https://dice-database.org/
CIS-BP Database (Build 2.00) Weirauch et al., 2014 http://cisbp.ccbr.utoronto.ca/TFTools.php
JASPAR Database (2020 release) Fornes et al., 2020 http://jaspar.genereg.net/
FIMO (v5.4.1) Grant et al., 2011 http://web.mit.edu/meme_v4.11.4/share/doc/fimo.html
HaploReg (v4.1) Broad Institute https://pubs.broadinstitute.org/mammals/haploreg/haploreg.php
UniPROBE Database (2019-03-03) Hume et al., 2015 http://thebrain.bwh.harvard.edu/uniprobe
ImageJ2 (v10.2) ImageJ Software https://imagej.net/ImageJ
IGV (V2.8.6) Thorvaldsdottir et al., 2013 http://www.igv.org/
MACS2 (v2.1.1) Zhang et al., 2008 https://github.com/taoliu/MACS
epiCOLOC (2020 release) Zhou et al., 2020 http://mulinlab.tmu.edu.cn/epicoloc
DeepBlue (2020 update) Albrecht et al., 2016 http://deepblue.mpi-inf.mpg.de
LDetect (first release) Berisa and Pickrell, 2016 http://bitbucket.org/nygcresearch/ldetect
gwas-pw (model 3) Pickrell et al., 2016 https://github.com/TankMermaid/gwas-pw
FINEMAP (v1.4) Benner et al., 2016 http://www.christianbenner.com/
RegulomeDB (v 1.1) Boyle et al., 2012 https://www.regulomedb.org/regulome-search/
regBase (v1.1.1) Zhang et al., 2019 https://github.com/mulinlab/regBase
GWAS4D (2019 update) Huang et al., 2018 http://mullinlab.tmu.edu.cn/gwas4d
gchromVAR (first release) Ulirsch et al., 2019 https://github.com/caleblareau/gchromVAR
GIGGLE (first release) Layer et al., 2018 https://github.com/ryanlayer/giggle
cepip (2018 release) Li et al., 2017b http://jjwanglab.org/cepip/
Cistrome DB (v1.1) Mei et al., 2017 http://cistrome.org/db/#/
ChromHMM (v1.20) Ernst and Kellis, 2017 http://compbio.mit.edu/ChromHMM/
DEPICT (v1_rel194) Pers et al., 2015 www.broadinstitute.org/depict
QTLtools (v1.1) Delaneau et al., 2017 https://qtltools.github.io/qtltools/
Basic4Cseq (v1.34) Walter et al., 2014 https://git.bioconductor.org/packages/Basic4Cseq
deepTools (v3.4.3) Ramirez et al., 2014 https://deeptools.readthedocs.io/
STAR (2.7.7a) Dobin et al., 2013 http://code.google.com/p/rna-star/
RSEM (v1.3.2) Li and Dewey, 2011 http://deweylab.biostat.wisc.edu/rsem
DESeq2 (v1.28) Love et al., 2014 http://www.bioconductor.org/packages/release/bioc/html/DESeq2.html
clusterProfiler (v3.8) Yu et al., 2012 http://bioconductor.org/packages/release/bioc/html/clusterProfiler.html
Roadmap Epigenomics Project (2015 release) Roadmap Epigenomics et al., 2015 http://www.roadmapepigenomics.org/
Bowtie2 (v2.2.1) Langmead and Salzberg, 2012 http://bowtie-bio.sourceforge.net/bowtie2/
BWA (v0.7.17) Li and Durbin, 2009 http://bio-bwa.sourceforge.net/
RStudio (version 1.0.136) with R (version 3.3.3) was used to analyze all statistical analyses in silico. GraphPad Prism version 8.1.1 software (La Jolla, CA) was used to analyze all experimental data. Data were shown as the means ± standard deviations (SD), "n" represents the number of biologically independent samples unless otherwise mentioned. An unpaired two-tailed Student's t test was used to define statistical significance for the experimental data analyses.

For manuscripts utilizing custom algorithms or software that are central to the research but not yet described in published literature, software must be made available to editors and reviewers. We strongly encourage code deposition in a community repository (e.g. GitHub). See the Nature Portfolio guidelines for submitting code & software for further information.

Data

Policy information about availability of data

All manuscripts must include a data availability statement. This statement should provide the following information, where applicable:

- Accession codes, unique identifiers, or web links for publicly available datasets
- A description of any restrictions on data availability
- For clinical datasets or third party data, please ensure that the statement adheres to our policy

All sequencing data generated in this study have been deposited in the Gene Expression Omnibus (GEO) database accession "GSE168045". The data generated in this study are provided in the Supplementary Information/Source Data file and based in human reference genome (GRCh37/hg19). The ATAC-seq profiles of hematopoietic and leukemic cell types, across 12 normal hematopoietic cell types data used in this study are available in the GEO database under accession "GSE74912 [https://www.ncbi.nlm.nih.gov/geo/query/acc.cgi]". Source data are provided with this paper.

Human research participants

Policy information about studies involving human research participants and Sex and Gender in Research.

Reporting on sex and gender	n/a
Population characteristics	n/a
Recruitment	n/a
Ethics oversight	n/a

Note that full information on the approval of the study protocol must also be provided in the manuscript.

Field-specific reporting

Please select the one below that is the best fit for your research. If you are not sure, read the appropriate sections before making your selection.

☒ Life sciences ☐ Behavioural & social sciences ☐ Ecological, evolutionary & environmental sciences

For a reference copy of the document with all sections, see nature.com/documents/nr-reporting-summary-flat.pdf

Life sciences study design

All studies must disclose on these points even when the disclosure is negative.

Sample size	Sample size were both indicated in the legend captions manuscript or the parametric results for all individuals for each sample plotted.
Data exclusions	No data were excluded from analyses.
Replication	Experiments described in the manuscript were performed at least twice as indicated in the figure legends unless otherwise mentioned. All attempts at replication were successful and all experimental findings were replicably reproduced.
Randomization	Samples were randomly allocated into experimental groups and control groups. There was no bias in the assignment of treatments.
Blinding	Investigators were blinded to experimental groups and control groups as data collection, and quantification was objective and not impacted by investigators presumptions.

Reporting for specific materials, systems and methods

We require information from authors about some types of materials, experimental systems and methods used in many studies. Here, indicate whether each material, system or method listed is relevant to your study. If you are not sure if a list item applies to your research, read the appropriate section before selecting a response.

Materials & experimental systems

n/a	Involved in the study	
☐	☒	Antibodies
☐	☒	Eukaryotic cell lines
☒	☐	Palaeontology and archaeology
☒	☐	Animals and other organisms
☒	☐	Clinical data
☒	☐	Dual use research of concern

Methods

n/a	Involved in the study	
☐	☒	ChIP-seq
☐	☒	Flow cytometry
☒	☐	MRI-based neuroimaging

Antibodies

Antibodies used

Antibody for ChIP-Seq
Rabbit polyclonal to ZBTB3, Novus Biologicals, Cat# NBP1-82079, Clone name: Polyclonal, RRID: AB_11029898, Dilution: 1:100
Rabbit monoclonal to CTCF, Abcam, Cat# ab128873, Clone name: EPR7314(B), RRID: AB_11144295, Dilution: 1:100
Rabbit polyclonal to RAD21, Abcam, Cat# ab992, Clone name: Polyclonal, RRID: AB_2176601, Dilution: 1:100
Anti-Rabbit IgG, Cell Signaling Technology, Cat# 7074, RRID: AB_2099233, Dilution: 1:100
Rabbit polyclonal to H3K27ac, Abcam, Cat# ab4729, RRID: AB_2118291, Dilution: 1:100
Rabbit monoclonal to H3K4me1, Cell Signaling Technology, Cat# 5326, RRID: AB_10695148, Dilution: 1:100
Rabbit monoclonal to H3K4me3, Cell Signaling Technology, Cat# 9751, RRID: AB_2616028, Dilution: 1:100
Antibody for Western blotting
Rabbit polyclonal to ZBTB3, Abcam, Cat# ab106536, Clone name: Polyclonal, RRID: AB_10863374, Dilution: 1:1000
Rabbit monoclonal to ACTB, Abclonal, Cat# AC026, Clone name: ARC5115-01, RRID: AB_2768234, Dilution: 1:50000
Rabbit polyclonal to GAPDH, Abclonal, Cat# AC001, Clone name: Polyclonal, RRID: AB_2619673, Dilution: 1:5000
Goat Anti Rabbit IgG-HRP, Thermo Fisher Scientific, Cat# G-21234, RRID: AB_1500696, Dilution: 1:5000

Validation

Antibodies were selected based on previous experience of the investigators and their use in the literature on human cells.
Rabbit polyclonal to ZBTB3, Novus Biologicals, Cat# NBP1-82079, RRID: AB_11029898, used for EMSA and ChIP, has been cited in the publication below.
Kottyan LC, Zoller EE, Bene J et al. The IRF5-TNPO3 association with systemic lupus erythematosus has two components that other autoimmune disorders variably share. Hum. Mol. Genet. 2014 Sep 08 [PMID: 25205108]

Rabbit monoclonal to CTCF, Abcam, Cat# ab128873, RRID: AB_11144295, used for ChIP-PCR and ChIP-seq, has been cited in the publications below.
Li Y et al. The structural basis for cohesin-CTCF-anchored loops. Nature 578:472-476 (2020). [PMID: 31905366]

Su QP et al. Superresolution imaging reveals spatiotemporal propagation of human replication foci mediated by CTCF-organized chromatin structures. Proc Natl Acad Sci U S A 117:15036-15046 (2020). [PMID: 32541019]
Ferrari R et al. TFIIIC Binding to Alu Elements Controls Gene Expression via Chromatin Looping and Histone Acetylation. Mol Cell 77:475-487.e11 (2020). [PMID: 31759822]

Rabbit polyclonal to RAD21, Abcam, Cat# ab992, RRID: AB_2176601, used for ChIP-PCR and ChIP-seq, has been cited in the publications below.
Luan J et al. Distinct properties and functions of CTCF revealed by a rapidly inducible degron system. Cell Rep 34:108783 (2021). [PMID: 33626344]
Laffleur B et al. Noncoding RNA processing by DIS3 regulates chromosomal architecture and somatic hypermutation in B cells. Nat Genet 53:230-242 (2021). [PMID: 33526923]

Anti-Rabbit IgG, Cell Signaling Technology, Cat# 7074, RRID: AB_2099233, used for ChIP-PCR and ChIP-seq, has been cited in the publications below.
Kelly E Leon et al. DOT1L modulates the senescence-associated secretory phenotype through epigenetic regulation of IL1A. Journal of Cell Biology, 2021. [PMID: 34037658]
Hongxu Xian et al. Metformin inhibition of mitochondrial ATP and DNA synthesis abrogates NLRP3 inflammasome activation and pulmonary inflammation. [PMID: 34115964]

Rabbit polyclonal to H3K27ac, Abcam, Cat# ab4729, RRID: AB_2118291, used for ChIP-PCR and ChIP-seq, has been cited in the publications below.
Wei J et al. Genome-wide CRISPR Screens Reveal Host Factors Critical for SARS-CoV-2 Infection. Cell 184:76-91.e13 (2021). [PMID: 33147444]
Yeola A et al. Induction of muscle-regenerative multipotent stem cells from human adipocytes by PDGF-AB and 5-azacytidine. Sci Adv 7:N/A (2021). [33523875]

Rabbit monoclonal to H3K4me1, Cell Signaling Technology, Cat# 5326, RRID: AB_10695148, used for ChIP-PCR and ChIP-seq, has been cited in the publications below.
Toshihiro Yoshie et al. Early high-fat feeding improves histone modifications of skeletal muscle at middle-age in mice. Laboratory Animal Research, 2020. [PMID: 32793459]
Angelika Feldmann et al. CDK-Mediator and FBXL19 prime developmental genes for activation by promoting atypical regulatory interactions. Nucleic Acids Res, 2020. [PMID: 31996894]

Rabbit monoclonal to H3K4me3, Cell Signaling Technology, Cat# 9751, RRID: AB_2616028, used for ChIP-PCR and ChIP-seq, has been cited in the publications below.
Iris Müller et al. MPP8 is essential for sustaining self-renewal of ground-state pluripotent stem cells. Nature Communications, 2021. [PMID: 34031396]
Mike R Wilson et al. ARID1A Mutations Promote P300-Dependent Endometrial Invasion through Super-Enhancer Hyperacetylation. Cell Rep, 2020. [PMID: 33176148]

Rabbit polyclonal to ZBTB3, Abcam, Cat# ab106536, RRID: AB_10863374, used for western blotting, has been cited in the publication below.
Jin X et al. SPOP targets oncogenic protein ZBTB3 for destruction to suppress endometrial cancer. Am J Cancer Res 9:2797-2812 (2019). [PMID: 31911863]

Rabbit monoclonal to ACTB, Abclonal, Cat# AC026, RRID: AB_2768234, used for western blotting, has been cited in the publications below.
Lu Yan et al. Maternal Huluwa dictates the embryonic body axis through β-catenin in vertebrates. Science, 2018 .[PMID: 30467143]
Liwen Zhang et al. Creatine promotes cancer metastasis through activation of Smad2/3. Cell metabolism, 2021. [PMID: 33811821]

Rabbit polyclonal to GAPDH, Abclonal, Cat# AC001, RRID: AB_2619673, used for western blotting, has been cited in the publications below.
Genschmer KR et al. Activated PMN Exosomes: Pathogenic Entities Causing Matrix Destruction and Disease in the Lung. Cell, 2019. [PMID: 30633902]
Jun Hu et al. A Microbiota-Derived Bacteriocin Targets the Host to Confer Diarrhea Resistance in Early-Weaned Piglets. Cell Host & Microbe, 2018. [PMID: 30543777]

Goat Anti Rabbit IgG-HRP, Thermo Fisher Scientific, Cat# G-21234, RRID: AB_1500696, used for western blotting, has been cited in the publications below.
Zhang XN et al. A ribose-functionalized NAD+ with unexpected high activity and selectivity for protein poly-ADP-ribosylation. Nature Communications, 2019. [PMID: 31519936]
Köhler AM et al. Integration of Fungus-Specific CandA-C1 into a Trimeric CandA Complex Allowed Splitting of the Gene for the Conserved Receptor Exchange Factor of CullinA E3 Ubiquitin Ligases in Aspergilli. mBio, 2019. [PMID: 31213557]

Eukaryotic cell lines

Policy information about cell lines and Sex and Gender in Research

Cell line source(s)	SC (cat# CRL-9855) and 293T (cat# CRL-3216) cells were purchased from the American Type Culture Collection (ATCC) (Virginia, USA). 293FT (cat# R70007) cells were purchased from Thermo Fisher Scientisic.
Authentication	Short tandem repeat authentication services from ATCC were used to authenticate the cell lines.
Mycoplasma contamination	The cell lines were regularly tested for mycoplasma and were found to be negative.

Commonly misidentified lines (See ICLAC register)	No commonly misidentified cell lines were used in this study.

ChIP-seq

Data deposition

☒ Confirm that both raw and final processed data have been deposited in a public database such as GEO.

☒ Confirm that you have deposited or provided access to graph files (e.g. BED files) for the called peaks.

Data access links *May remain private before publication.*	The accession number for SC H3K27ac, H3K4me1, H3K4me3, CTCF, ZBTB3, and RAD21 ChIP-seq, reported in this paper is GEO: GSE168045. There is no restrictions on data availability.
Files in database submission	GSM5124337 SC cells-H3K27ac-ChIP-seq<br>GSM5124338 SC cells-H3K4me1-ChIP-seq<br>GSM5124339 SC cells-H3K4me3-ChIP-seq<br>GSM5124341 SC cells-Input-ChIP-seq<br>GSM5124342,GSM6632505,GSM6632506 SC cells-ZBTB3-ChIP-seq with three replicates<br>GSM5124340,GSM6632507,GSM6632508 SC cells-RAD21-ChIP-seq with three replicates<br>GSM5124343 SC cells-CTCF-ChIP-seq
Genome browser session (e.g. UCSC)	Not applicable.

Methodology

Replicates	ChIP-seq experiments for ZBTB3 and RAD21 were performed for three biological replicates, and our findings were validated by ChIP-qPCR and Sanger sequencing.
Sequencing depth	ChIP-seq were sequenced 150bp paired-ends with at least 20 million reads each sample.
Antibodies	Rabbit polyclonal to ZBTB3, Novus Biologicals, Cat# NBP1-82079, Clone name: Polyclonal, RRID: AB_11029898, Dilution: 1:100<br>Rabbit monoclonal to CTCF, Abcam, Cat# ab128873, Clone name: EPR7314(B), RRID: AB_11144295, Dilution: 1:100<br>Rabbit polyclonal to RAD21, Abcam, Cat# ab992, Clone name: Polyclonal, RRID: AB_2176601, Dilution: 1:100<br>Anti-Rabbit IgG, Cell Signaling Technology, Cat# 7074, RRID: AB_2099233, Dilution: 1:100<br>Rabbit polyclonal to H3K27ac, Abcam, Cat# ab4729, RRID: AB_2118291, Dilution: 1:100<br>Rabbit monoclonal to H3K4me1, Cell Signaling Technology, Cat# 5326, RRID: AB_10695148, Dilution: 1:100<br>Rabbit monoclonal to H3K4me3, Cell Signaling Technology, Cat# 9751, RRID: AB_2616028, Dilution: 1:100
Peak calling parameters	All the samples were separately processed, and the unique mapped reads were kept. The peaks were called by MACS2 (v2.1.1) (Zhang et al., 2008) with default parameters using IgG as control. Enrichment was calculated.
Data quality	PCR duplicates were removed.
Software	For data analysis, the sequences with adaptor and low quality were trimmed for all 150-bp pair-end reads. The clean reads were mapped against the human reference genome (GRCh37/hg19) using BWA (Li and Durbin, 2009). All the samples were separately processed, and the unique mapped reads were kept. The peaks were called by MACS2 (v2.1.1) (Zhang et al., 2008) using IgG as control. The results were analyzed and visualized in IGV (v2.8.6) (Thorvaldsdottir et al., 2013).

Flow Cytometry

Plots

Confirm that:

☒ The axis labels state the marker and fluorochrome used (e.g. CD4-FITC).

☒ The axis scales are clearly visible. Include numbers along axes only for bottom left plot of group (a 'group' is an analysis of identical markers).

☒ All plots are contour plots with outliers or pseudocolor plots.

☒ A numerical value for number of cells or percentage (with statistics) is provided.

Methodology

Sample preparation	Human peripheral blood monocyte-derived SC cell line (American Type Culture Collection [ATCC], CRL-9855) was cultured in Iscove's Modified Eagle's Medium (IMDM; ThermoFisher, 31980030) containing 10% Fetal Bovine Serum (FBS; ThermoFisher, 16140071). The SC-derived cells were resuspended in fresh medium with a density of 1E+07/ml, and then gated and sorted by Flow cytometry.
Instrument	Becton, Dickinson and Company, BD FACSAria II

Software	We used FlowJo (V10.7) (Roca et al., 2021) to collect and analyze the flow cytometry data.
Cell population abundance	We used the pure SC cell line to gating and sorting, and the abundance of the GFP-positive SC-derived cells within post-sort fractions is nearly 100 . We observed and identified it under fluorescence microscope.
Gating strategy	For all genome editing assays, 1E+04 SC cells were collected for gating, we discarded cell fragments, dead cells and adherent cells, and gated the single live SC cells according to the size. We used the SC cells without GFP expression as control, and sorted the single GFP-positive SC-derived cells into 96-well plates. After expending culture of the sorted cells from 96-well plates to 24-well plates, genomes of the cells were separately extracted for genotyping. Strategy for the flow cytometry gating and sorting in genome editing was shown in Supplementary Fig. 11.

☒ Tick this box to confirm that a figure exemplifying the gating strategy is provided in the Supplementary Information.
